# Time Course of Metabolic, Neuroendocrine, and Adipose Effects During 2 Years of Follow-up After Gastric Bypass in Patients With Type 2 Diabetes

**DOI:** 10.1210/clinem/dgab398

**Published:** 2021-06-04

**Authors:** Kristina E Almby, Petros Katsogiannos, Maria J Pereira, F Anders Karlsson, Magnus Sundbom, Urban Wiklund, Prasad G Kamble, Jan W Eriksson

**Affiliations:** 1 Department of Medical Sciences, Uppsala University, Uppsala, Sweden; 2 Department of Surgical Sciences, Uppsala University, Uppsala, Sweden; 3 Department of Radiation Sciences, Umeå University, Umeå, Sweden

**Keywords:** T2D, RYGB, neuroendocrine changes, adipose effects

## Abstract

**Context:**

Roux-en-Y gastric bypass surgery (RYGB) markedly improves glycemia in patients with type 2 diabetes (T2D), but underlying mechanisms and changes over time are incompletely understood.

**Objective:**

Integrated assessment of neuroendocrine and metabolic changes over time in T2D patients undergoing RYGB.

**Design and Setting:**

Follow-up of single-center randomized study.

**Patients:**

Thirteen patients with obesity and T2D compared to 22 healthy subjects.

**Interventions:**

Blood chemistry, adipose biopsies, and heart rate variability were obtained before and 4, 24, and 104 weeks post-RYGB.

**Results:**

After RYGB, glucose-lowering drugs were discontinued and hemoglobin A1c fell from mean 55 to 41 mmol/mol by 104 weeks (*P* < 0.001). At 4 weeks, morning cortisol (*P* < 0.05) and adrenocorticotropin (*P* = 0.09) were reduced by 20%. Parasympathetic nerve activity (heart rate variability derived) increased at 4 weeks (*P* < 0.05) and peaked at 24 weeks (*P* < 0.01). C-reactive protein (CRP) and white blood cells were rapidly reduced (*P* < 0.01). At 104 weeks, basal and insulin-stimulated adipocyte glucose uptake increased by 3-fold *vs* baseline and expression of genes involved in glucose transport, fatty acid oxidation, and adipogenesis was upregulated (*P* < 0.01). Adipocyte volume was reduced by 4 weeks and more markedly at 104 weeks, by about 40% *vs* baseline (*P* < 0.01).

**Conclusions:**

We propose this order of events: (1) rapid glucose lowering (days); (2) attenuated cortisol axis activity and inflammation and increased parasympathetic tone (weeks); and (3) body fat and weight loss, increased adipose glucose uptake, and whole-body insulin sensitivity (months-years; similar to healthy controls). Thus, neuroendocrine pathways can partly mediate early glycemic improvement after RYGB, and adipose factors may promote long-term insulin sensitivity and normoglycemia.

Bariatric surgery is currently the most effective treatment for obesity and its metabolic comorbidities (1-3). It is often referred to as “metabolic surgery” (4) and is typically performed in patients with body mass index (BMI) > 35kg/m^2^, but comorbidities such as type 2 diabetes may lower this cutoff (5). Roux-en-Y gastric bypass (RYGB) has been the most common procedure in the last decades, and it results in about 60% average loss of excess body weight (6,7). Before weight loss, there is commonly an improvement of glycemic control. This is initially due to reduced hepatic glucose production and thereafter improved insulin sensitivity, and gut hormone responses are also of importance (1). The metabolic improvements are clinically significant, and patients with type 2 diabetes can often discontinue their antidiabetic treatment shortly after surgery (8,9). In obese patients without diabetes, the risk for future type 2 diabetes is markedly reduced (2). The diabetes-related improvements are not strictly correlated with the degree of weight loss and are also seen in mild obesity (10).

The mechanisms underlying the antidiabetic effects of RYGB are not completely understood. Several studies have shown enhanced responses of gut-derived incretin hormones (ie, glucagon-like peptide-1 and glucose-dependent insulinotropic polypeptide) (11). Other gut hormones [eg, PYY and oxyntomodulin (12,13)] have also been implicated and so have changes in bile acid secretion, gut microbiota, and gastrointestinal motility (14). Some data suggest that autonomic nervous system (ANS) dysfunction may be present in obesity (15), and previous research has observed signs of altered ANS activity after RYGB as reflected by heart rate variability (HRV) assessments (16,17).

Adipose tissue is a well-established endocrine organ, affecting whole-body energy metabolism largely via release of adipokines, cytokines, energy substrates, and other biomolecules (18). Following bariatric surgery, changes to adipose tissue volume, distribution, and cellular functions are observed (19). It is not known to what extent these changes contribute to the metabolic improvements following bariatric surgery.

We previously published 4- and 24-week results of the present study in patients with type 2 diabetes and obesity who were randomly assigned to RYGB or usual care (ie, medication and life style advice) (16,20). After RYGB, there were early changes in ANS activity and lowering of morning cortisol levels as well as reduction in fat cell size. This was followed by functional changes in adipose tissue gene expression and glucose uptake. The aim of this report is to address metabolic and neuroendocrine changes as they occur over a longer, 2-year time period after RYGB. In addition, new analyses on systemic inflammation are performed. We provide integrated and repeated assessments of several pathways involving hormonal, inflammatory, ANS, and adipose functions and propose a chain of events contributing the antidiabetic actions of RYGB. In addition, participants at 104 weeks’ post-RYGB were compared to a healthy BMI-matched control group to address whether they partly, completely, or exceedingly normalize adipose and whole-body metabolism in relation to their new lower body weight.

## Materials and Methods

### Participants and Protocol

The study design was previously described in detail (16,20). Patients were approached in connection with a visit to the obesity outpatient clinic at the Department of Endocrinology and Diabetes at Uppsala University Hospital in Sweden. Inclusion criteria were 18 to 60 years old, a BMI of 30 to 45 kg/m^2^ with type 2 diabetes for a maximum of 10 years, and treated with maximally 3 antidiabetic agents but not insulin. Patients were randomized to either RYGB surgery or usual care in a 2:1 ratio. The usual care group was followed for 24 weeks, when some of them opted for surgery. Their data were previously presented in detail (16), and herein we report long-term results for the RYGB group. Investigation visits were as follows: an enrollment visit at baseline; after an initial 4-week period with low-calorie diet (LCD; 800-1100 kcal/day according to routine) on the day of RYGB surgery; and at 4 weeks, 24 weeks, and 104 weeks after surgery. The RYGB surgery was performed laparoscopically at the Department of Surgery at Uppsala University Hospital. The main results from the 4- and 24-week visits and the limited data after LCD are previously reported (16,20). Initially, 11 females and 3 males were included in the RYGB surgery group; mean age was 51 years at the start of the study. One female subject was excluded after the first visit due to being diagnosed with an eating disorder, and another female subject did not undergo the last visit due to pregnancy. Thus, results are reported for 13 subjects in total but for 12 at 104 weeks. For further evaluation of clinical and adipose tissue effects after RYGB, we included a group of nondiabetic, healthy individuals who donated subcutaneous adipose tissue (n = 22), and they were matched to post-RYGB patients (at 104 weeks) for age, sex, and BMI.

During all visits, resting blood pressure, fasting blood samples, and anthropometric data were obtained, and body composition was assessed with bioimpedance (Tanita body composition analyzer, BC-418). An electrocardiogram recording for HRV (21) analysis was performed during rest for 6 minutes using a single-channel system, developed at the Umeå University Hospital, Sweden. At baseline, 4, 24, and 104 week visits a subcutaneous adipose tissue biopsy from the abdominal area and arginine and oral glucose tolerance tests (OGTTs) were performed as previously reported (16).

### Heart Rate Variability Analysis

HRV is based on the oscillations in heart rate between successive beats (ie, changes in the R-R intervals in the electrocardiogram) (22). Spectral analysis of HRV analysis determines the power (signal energy) or the strength of the variation of specific frequency components (23).

Total spectral power, the power of the low-frequency (P_LF_; 0.04-0.15 Hz), and high-frequency (P_HF_; 0.15-0.50 Hz) domains, all log-transformed, were analyzed, using MATLAB (MathWorks, Natick, MA, USA). The method of data extraction from the recorded electrocardiograms has been previously specified (24).

### Blood Chemistry

Blood samples were drawn after an overnight fast from the antecubital vein and analyses were performed at the Uppsala University Hospital Clinical Chemistry facility. Glucose was determined with the Architect assay (Abbott, North Chicago, IL, USA). Insulin, cortisol, and C-peptide were determined using Cobas e (Roche, Indianapolis, IN, USA), insulin-like growth factor 1 (IGF-1) with Liaison XL (DiaSorin, Saluggia, Italy) and adrenocorticotropin (ACTH) with Immulite 2000XPi (Siemens Healthcare Global, Erlangen, Germany). Free fatty acids (FFAs) and glycerol were measured using enzymatic assays as previously reported (16,20). All protocols were followed according to the manufacturer’s instructions.

### Subcutaneous Adipose Tissue Assessments

Subcutaneous adipose tissue was collected from the abdomen through needle aspiration under local anesthesia with lidocaine at baseline and at 4, 24, and 104 weeks post-RYGB, and through a laparoscopic incision site on the day of surgery. Some adipose tissue was snap-frozen in liquid nitrogen for gene expression analysis, whereas the other part was used to perform ex vivo metabolic assays Adipocytes were isolated after adipose tissue digestion with collagenase A. Basal and insulin-stimulated glucose uptake rate was assessed as ^14^C-D-glucose uptake into adipocytes. Basal or hormone-regulated lipolysis rate was measured as glycerol release from adipocytes. Adipocyte size was determined with standardized light microscopy in cell monolayers. All such analyses of adipose gene expression and adipocyte metabolism were performed as previously reported in detail (20,25).

### Statistical Analyses

The sample size was chosen, based on previous work (26), to provide at least 80% power to detect changes of the formal primary endpoint hemoglobin A1c (HbA1c) of ≥1% unit after RYGB. The final sample size of 13 RYGB patients allows detection of 15% changes to adipose measures such as glucose uptake and lipolysis with 80% power.

Data that were not normally distributed when assessed with Shapiro-Wilks test were log-transformed. Clinical, biochemical, hormonal, and adipocyte data were analyzed with a mixed-effects model for repeated measures with pairwise comparisons between the baseline and the respective postoperative visits. The Benjamini-Hochberg method was used to control for false discovery rate with multiple comparisons.

The comparison between controls and 104 weeks was done using unpaired *t*-test. Data are presented as mean (SD or SEM as specified) unless otherwise is indicated. *P*-values < 0.05 were considered to be statistically significant.

Adipose gene expression was normalized to baseline, and relative changes are reported. For hormonal and metabolic measures additional mixed effect modeling was used including the baseline level as a covariate.

Analyses were performed in Microsoft Excel (Microsoft Corporation, Redmond, WA, USA), GraphPad Prism 8.0 (GraphPad Software, La Jolla, CA, USA), and SPSS (IBM, Armonk, NY, USA).

### Ethics

All subjects received verbal and written information prior to signing an informed consent form. The study was conducted in accordance with the Declaration of Helsinki and approved by the Ethics Committee of Uppsala (Dnr 2014/255). The matched healthy control subjects were participants in a study focused on metabolic regulation in human adipose tissue (Dnr 2018/385, 2013/494).

## Results

### Change in Medications

Twelve subjects were on antidiabetic treatment at the first visit. At 4 weeks post-RYGB, all patients except 1 had discontinued their antidiabetic treatment. The patient who continued metformin treatment received a lower dose. At 24 weeks and 104 weeks, 2 and 4 subjects, respectively, were treated with antidiabetic agents. Ten subjects were on lipid-lowering treatment before surgery, but at 4 weeks and 24 weeks, all had discontinued lipid-lowering treatment. At 104 weeks, 3 subjects had lipid-lowering treatment. Eight subjects had 1 or more antihypertensive drugs at baseline. After RYGB, all but 1 had discontinued medication at 4 weeks, and all, by 24 weeks. At 104 weeks, 1 subject had resumed antihypertensive medication.

### Effects on Body Composition and Glucose and Lipid Metabolism

**Figure 1.**
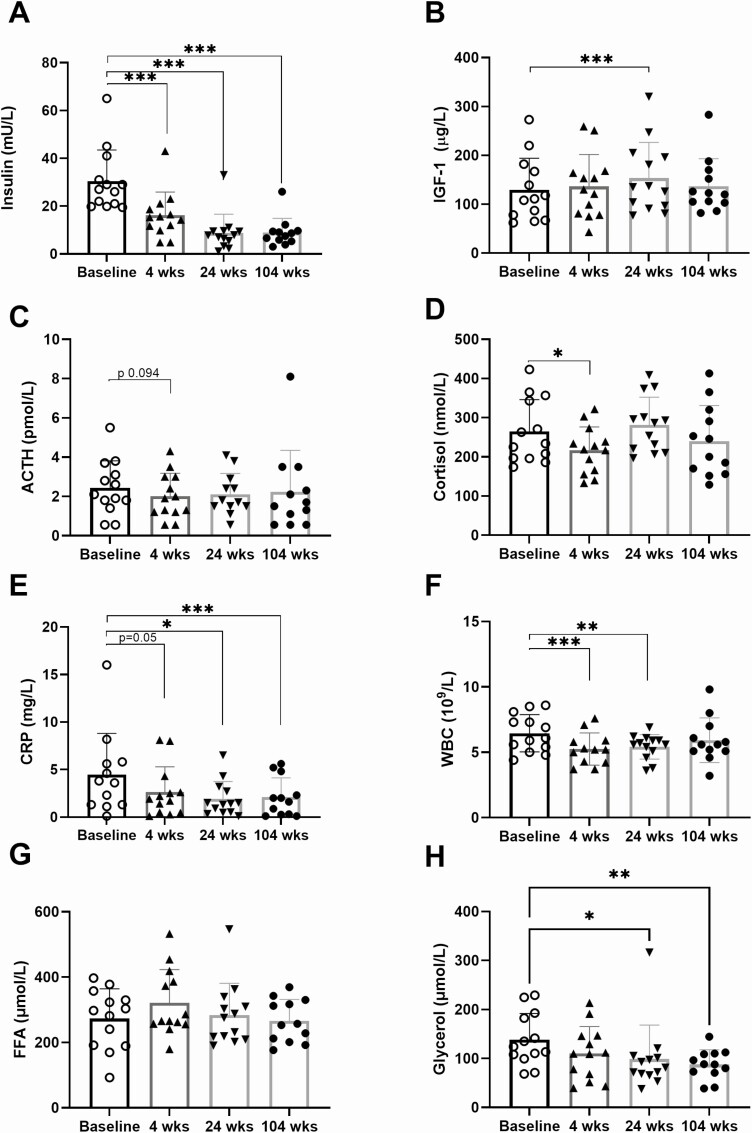
Inflammatory, hormonal and lipolysis measures. Fasting morning levels of (A) insulin, (B) IGF-1, (C) ACTH, (D) cortisol, (E) CRP, (F) WBC, (G) FFAs, and (H) glycerol at baseline and at 4, 24, and 104 weeks after RYGB. Data are expressed as means ± SD. *P*-values from pairwise comparison with baseline, after using mixed effects model, corrected for false detection rate. *P < 0.05, ***P < 0.01, ***P < 0.001.

Results are summarized in Table 1. Weight, BMI, waist and hip circumference, total body fat, HbA1c, and fasting glucose were reduced at 4 and 24 weeks and remained lower than baseline at 104 weeks. The Matsuda index was increased at 4 and 24 weeks, and although it decreased somewhat at 104 weeks, it remained significantly higher than baseline. Accordingly, homeostatic model assessment for insulin resistance changed in the inverse direction. The decrease in total cholesterol and low-density lipoprotein seen at 4 weeks was not present at 24 and 104 weeks, whereas high-density lipoprotein was elevated at 24 and 104 weeks, and fasting triglycerides remained reduced at all time points. At 104 weeks, only HbA1c and glucose levels were significantly but only slightly elevated compared to the BMI-matched healthy control group. Plasma FFA area under the curve (AUC) during OGTT was reduced at 24 and 104 weeks compared to baseline and glycerol AUC during the OGTT was reduced at 104 weeks (Table 1). Fasting FFA levels did not change during the visits, but fasting glycerol was reduced at 24 and 104 weeks compared to baseline (Fig. 1G and 1H).

**Table 1.** Anthropometric and metabolic characteristics of patients before and after RYGB and of control subjects

	Baseline	4 weeks	24 weeks	104 weeks	Controls*a*
Sex (males/females, n)	3M/10F	3M/10F	3M/10F	3M/9F	6M/16F
Age (years)	51 (10)	—	—	55 (7)	55 (10)
Weight (kg)	99.8 (13.7)	89.0 (10.9)^***^	77.3 (9.5)^***^	76.9 (10.1)^***^	81.2 (11.2)
BMI (kg/m^2^)	36.8 (3.9)	32.8 (3.2)^***^	28.5 (3.2)^***^	28.6 (3.5)^***^	28.3 (3.5)
Waist circumference (cm)	114.5 (6.2)	106.0 (6.5)^***^	96.5 (7.0)^***^	95.8 (8.8)^***^	97.1 (11.7)
Hip circumference (cm)	117.5 (8.4)	110.5 (7.6)^***^	103.2 (5.2)^***^	102.6 (7.1)^***^	107.3 (7.2)
Waist/Hip ratio	1.0 (0.1)	1.0 (0.1)	0.9 (0.1)*	0.9 (0.1)^**^	0.9 (0.1)
Total body fat (%)	42.8 (7.5)	40.0 (7.5)^***^	33.1(9.0)^***^	33.7 (9.3)^***^	31.8 (6.6)
BP systolic (mmHg)	136 (14)	126 (11)*	132 (17)	136 (24)	135 (19)
BP diastolic (mmHg)	79 (11)	75 (6)	77 (8)	79 (12)	74 (11)
HbA1c (mmol/mol)	54.9 (12.2)	46.2 (12.9)^***^	40.5 (5.9)^***^	41.4 (5.2)^***^	34.7 (3.4)^***,*b*^
HbA1c (% NGSP)	7.2 (1.1)	6.4 (1.1)^***^	5.9 (0.5)^***^	6.0 (0.4)^***^	5.4 (0.3)^***,*b*^
P-glucose (mmol/L)	8.3 (1.9)	7.1 (1.8)^**^	6.3 (1.3)^***^	6.5 (1.1)^**^	5.8 (0.6)^*,*b*^
Matsuda index	1.4 (0.5)	2.8 (1.2)^***^	4.2 (1.7)^***^	3.8 (1.9)^**^	NA
FFA AUC (min × µmol/L)	27920 (9709)	27088 (9804)	20561 (6083)^**^	13583 (4771)^***^	NA
Glycerol AUC (min x µmol/L)	21207 (14217)	17220 (12240)	17549 (14082)	10862 (4377)*	NA
HOMA-IR	8.9 (4.5)	3.3 (2.0)^***^	1.6 (0.6)^***^	2.2 (1.3)^***^	2.4 (1.4)
Insulinogenic index	2.0 (1.5)	3.3 (4.2)	2.3 (2.3)	2.2 (1.0)	NA
P-cholesterol (mmol/L)	4.7 (0.9)	3.9 (1.0)^***^	4.3 (1.0)	4.7 (1.0)	5.7 (1.0)^**,*b*^
P-HDL-cholesterol (mmol/L)	1.0 (0.1)	0.9 (0.2)^**^	1.1 (0.2)^**^	1.1 (0.1)^**^	1.4 (0.3)^**,*b*^
P-LDL-cholesterol (mmol/L)	2.9 (0.8)	2.5 (0.9)*	2.7 (0.9)	3.1 (1.1)	3.7 (0.9)
fP-triglycerides (mmol/L)	2.2 (0.9)	1.4 (0.4)^**^	1.2 (0.3)^***^	1.2 (0.4)^**^	1.2 (0.6)
B-White blood cell count (10^9^/L)	6.5 (1.4)	5.2(1.2)^***^	5.4(0.9)^**^	5.9 (1.7)	5.1 (0.9)
P-C reactive protein (mg/L)	4.5 (4.3)	2.6 (2.7)	1.9 (1.8)^**^	2.1 (2.0)*	2.0 (1.9)

Anthropometric and metabolic characteristics at baseline and at 4, 24, and 104 weeks after gastric bypass surgery. Matsuda and AUC for FFA and glycerol are from 180 min OGTT. Data are means (SD). *P*-values represent pairwise comparison between each postoperative visit and baseline, corrected for false discovery rate. *P < 0.05, **P < 0.01, ***P < 0.001.

Abbreviations: B, whole blood; BP, blood pressure; f, fasting; HDL, high-density lipoprotein; HOMA-IR, homeostatic model assessment for insulin resistance; LDL, low-density lipoprotein; P, plasma.

^*a*^Control group of healthy subjects, matched for BMI, age, and sex to patients 104 week post-RYGB.

^*b*^Significant differences between controls and 104 weeks post-RYGB.

### Inflammation and Hormone Levels

CRP, white blood cells (WBCs), and hormone levels were analyzed in fasting morning samples and are shown in Figure 1. CRP levels were markedly and significantly lowered compared to baseline at 4 weeks post-RYGB, and they were further reduced at 24 weeks and then remained stable at 104 weeks. The WBC count was reduced from 4 weeks and onward. ACTH was numerically reduced at 4 weeks and 24 weeks relative to baseline. Morning serum cortisol was significantly lowered from baseline by about 20% at 4 weeks but not at 24 or 104 weeks. Serum insulin was significantly reduced relative to baseline at all time points. IGF-1 was significantly increased at 24 weeks.

### Blood Pressure, Heart Rate, and Heart Rate Variability

Systolic blood pressure was lower at 4 weeks but gradually returned to baseline levels at 104 weeks (Table 1). Diastolic blood pressure showed a similar trend, and there was no change in diastolic blood pressure at 104 weeks. In reality, there were however more marked and sustained blood-pressure lowering effects, since this was masked by discontinuation of antihypertensives by all patients. Clinical guidelines were followed aiming to maintain blood pressure below 140/85 mmHg.

Data from resting HRV recordings are shown in Table 2. Recordings from 1 subject were excluded due to arrhythmia. The analyses showed an increase in the R-R interval at 4 weeks, which remained significantly increased at 24 and 104 weeks. Total spectral power and the 2 spectral components P_LF_ and P_HF_ were also significantly increased at 4 and 24 weeks but not at 104 weeks. The P_LF_-to-P_HF_ ratio was reduced at all visits, although significantly only at 24 weeks.

**Table 2.** Heart rate variability measures: post-RYGB change from baseline

		Change from baseline		
Measure	Baseline	4 weeks	24 weeks	104 weeks
R-R (s)	0.80 (0.04)	+0.16 (0.09)*	+0.18 (0.07)^***^	+0.13 (0.14)*
P_tot_ (ms^2^,log)	2.98 (0.14)	+0.22 (0.21)*	+0.36 (0.35)^**^	+0.13 (0.36)
P_VLF_ (ms^2^,log)	2.65 (0.14)	+0.18 (0.30)	+0.28 (0.38)*	+0.14 (0.50)
P_LF_ (ms^2^,log)	2.48 (0.14)	+0.21 (0.24)*	+0.30 (0.40)*	+0.13 (0.34)
P_HF_ (ms^2^,log)	2.10 (0.19)	+0.29 (0.26)*	+0.59 (0.46)^**^	+0.18 (0.39)
P_LF/PHF_	0.37 (0.09)	−0.09 (0.29)	−0.28 (0.30)*	−0.05 (0.24)

Data are means (SD) for baseline and changes at follow-up, respectively (n = 10). Spectral indices are log-transformed. *P*-values refers to pairwise comparisons with baseline. *P < 0.05, **P < 0.01, ***P < 0.001.

Abbreviations: P_HF_, power of high frequency component; P_LF_, power of low frequency component; P_tot_, total power; P_VLF_, power of very low frequency component; R-R, mean R-R interval.

Neither baseline BMI or body weight loss was significantly associated with changes in HbA1c, the Matsuda index, or any of the HRV, hormonal, and inflammatory measures as previously reported. Additional analyses using mixed-effect modeling, including baseline levels of blood chemistry measures, overall confirmed the reported post-RYGB effects.

### Adipocyte Morphology

**Figure 2.**
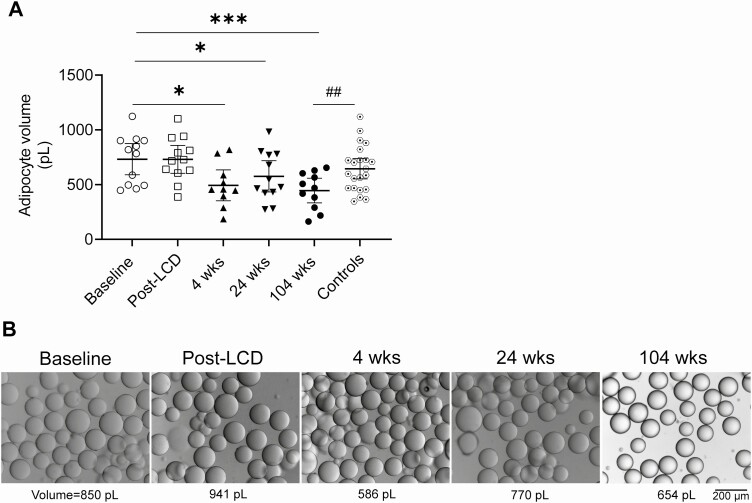
Adipocyte morphology after RYGB. (A) Average adipocyte volumes shown in picoliters (pL; means ± SEM, horizontal lines) for baseline, post-LCD, 4 weeks, 24 weeks, 104 weeks and controls, respectively (n = 12, 12, 10, 12, 11, and 22, respectively). *P < 0.05, ***P < 0.001 *vs* baseline; ^##^*P* < 0.01 for controls *vs* 104 weeks. (B) Representative images of adipocyte size from 1 subject at different study visits. All images and measurements were obtained using a light microscope with a magnification of ×100.

Adipocyte volume at baseline and post-LCD was similar, but at all postsurgery visits, it was significantly reduced compared to baseline (Fig. 2). Furthermore, the average adipocyte volume was about 30% lower in patients at 104 weeks postsurgery when compared to the BMI-matched control subjects (446 ± 168 pL *vs* 645 ± 209 pL, *P* < 0.01). A higher proportion of smaller adipocytes was seen over time after surgery, and the distribution curve was clearly shifted toward smaller sizes at 104 weeks (data not shown).

### Adipocyte Glucose Uptake and Lipolysis

**Figure 3.**
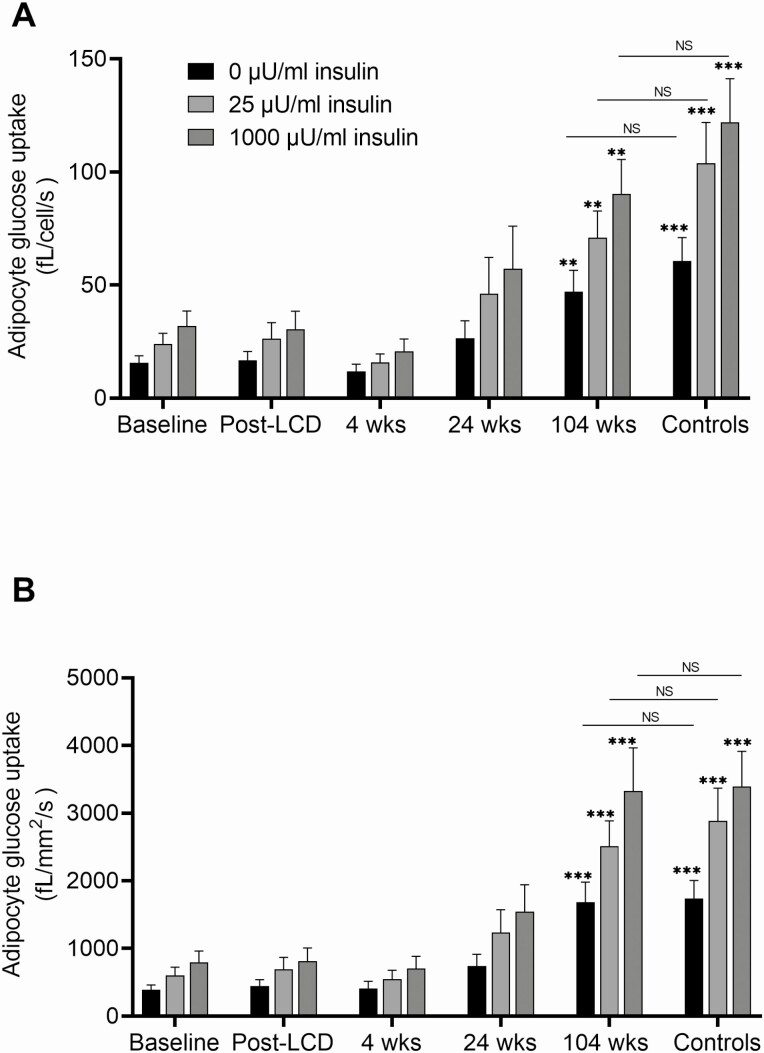
Adipocyte glucose uptake after RYGB. Basal and insulin-stimulated glucose uptake in isolated adipocytes. (A) Adjusted for number of cells; (B) adjusted for cell surface area. Data are shown as means ± SEM. **P < 0.01; ***P < 0.001 *vs* baseline. Abbreviation: NS, nonsignificant for controls *vs* 104 weeks.

**Figure 4.**
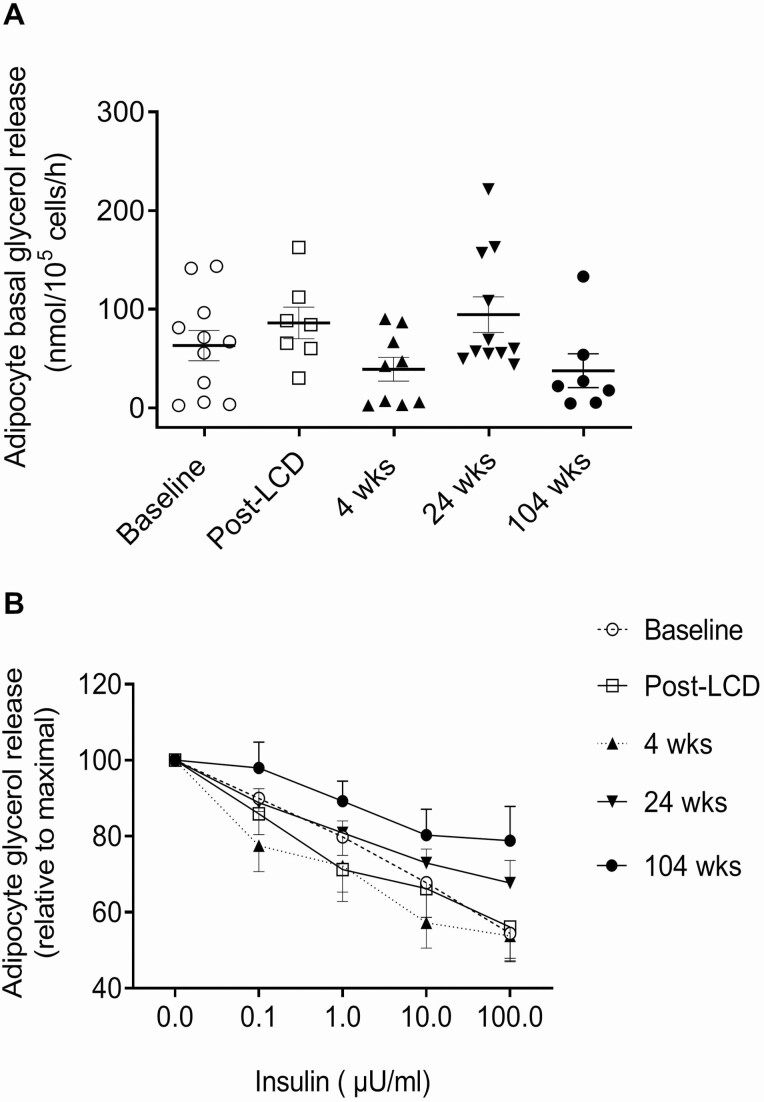
Adipocyte lipolysis after RYGB. (A) Isolated adipocytes were incubated without hormones or (B) with isoproterenol 0.5 µM together with different concentrations of insulin (0-100 µU/mL), and glycerol release was measured. Data are means ± SEM, and there were no significant differences (baseline *vs* post-LCD, 4 weeks, 24 weeks, and 104 weeks postsurgery; n = 12, 7, 10, 12, and 8, respectively, due to limited tissue amounts).

The basal and insulin-stimulated glucose uptake is shown in Figure 3. It was similar for baseline *vs* post-LCD 4 weeks post-RYGB visits. At 24 weeks post-RYGB, glucose uptake tended to rise and at 104 weeks, basal as well as submaximal and maximal insulin-stimulated glucose uptake, was significantly higher than baseline, and this was seen when calculated per cell and, in particular, per cell surface area. Moreover, the basal and insulin-stimulated glucose uptake at 104 weeks was comparable to that of glucose uptake in adipocytes from the age, sex, and BMI-matched controls (Fig. 3A). The relative response to insulin did not change between baseline and postsurgery visits (data not shown). No significant change was seen in basal, isoproterenol-stimulated, or insulin-inhibited lipolysis in adipocytes at any post-RYGB visit compared to baseline (Fig. 4; data not shown).

### Adipose Tissue Gene Expression

The data are summarized in Table 3. We studied the expression of genes that are involved in insulin action, cell proliferation, adipogenesis and lipid metabolism, glucose transport, mitochondrial fatty acid oxidation, and pro- or anti-inflammatory cytokines. The data up to 24 weeks for some of the genes are previously reported (20). The expression of genes for adiponectin and leptin increased at 104 weeks post-RYGB relative to baseline, whereas the resistin gene expression was decreased at all visits after surgery compared to baseline. Genes involved in adipogenesis (*PPARG*, *CEBPA*, *CEBPB*, *BMP4*, *FABP4*, and *FAS*), glucose transport (*GLUT1*, *GLUT4*, *AKT1*, and *IRS1*), and fatty acid oxidation (*CPT1B)* were increased at 104 weeks compared to baseline. The *IL33* gene expression increased nonsignificantly at postsurgery visits compared to baseline (data not shown). The expression of *IL18* was reduced at 104 weeks postsurgery. The expression of genes regulated by glucocorticoids such as *CNR1*, *LCN2*, and *FKBP5* was increased at postsurgery visits.

**Table 3.** Fold change in adipose gene expression postsurgery compared to baseline

Function/gene symbol	4 weeks (n = 11)	24 weeks (n = 12)	104 weeks (n = 10)
Adipokines			
* LEP*	0.75 (0.12)^**^	1.91 (0.40)	2.63 (0.64)*
* ADIPOQ*	4.81 (2.72^) #^	12.15 (5.60)^**^	3.3 (0.96)*
* RESTN*	0.73 (0.15)*	0.50 (0.07)^***^	0.67 (0.13)^**^
* NAMPT*	1.00 (0.07)	1.13 (0.06) ^#^	1.00 (0.08)
Cell proliferation			
* E2F1*	0.71 (0.06)^**^	0.67 (0.11)^**^	1.49 (0.27) ^#^
Adipogenesis and lipid metabolism			
* PPARG*	1.22 (0.24)	2.14 (0.42)^**^	0.91 (0.15)
* CEBPA*	1.06 (0.17)	1.88 (0.44)	1.93 (0.40)*
* CEBPB*	1.24 (0.20)	1.64 (0.23)*	2.63 (0.27)^***^
* FAS*	1.76 (0.54)	5.67 (1.84)*	6.54 (1.75)^**^
* BMP4*	0.70 (0.20)*	1.20 (0.50)	6.81 (2.04)^**^
* FABP4*	2.70 (0.70)*	2.91 (0.72)*	0.88 (0.31) ^#^
Mitochondrial function			
* CPT1B*	1.53 (0.13)^**^	2.13 (0.32)^**^	3.18 (0.43)^***^
Glucose transport			
* SLC2A1*	1.15 (0.15)	1.28 (0.16)	1.77 (0.36)
* SLC2A4*	1.32 (0.31)	3.57 (1.11)^**^	2.41 (0.77)^**^
* IRS1*	1.20 (0.18)	1.66 (0.26)*	1.71 (0.44)^#^
* AKT1*	1.21 (0.11)	1.37 (0.11)^**^	2.00 (0.15)^***^
Pro/anti-inflammatory cytokines			
* IL6*	0.91 (0.15)	0.73 (0.11)*	0.92 (0.14) ^#^
* IL18*	0.81 (0.08)*	0.51 (0.07)^***^	0.46 (0.11)^**^
Glucocorticoid-regulated genes			
* FKBP51*	1.36 (0.20)	2.75 (0.78)^**^	1.58 (0.34)
* CNR1* ^*a*^	2.86 (0.86)	4.91 (1.70)*	3.67 (1.61)
* LCN2*	2.75 (1.04)	3.44 (1.29)	20.48 (17.03)*
11HSD1B*a*	1.21 (0.18)	0.99 (0.15)	1.16 (0.17)

Data are means (SEM) and show as fold changes at 4 weeks, 24 weeks, and 104 weeks after RYGB relative to baseline. (values < 1 = decrease, >1 = increase in gene expression after RYGB *vs* baseline). All data were log transformed before statistical analysis; n = 10, 11, and 9 for visits at 4, 24, and 104 weeks, respectively. *P < 0.05, **P < 0.01, ***P < 0.001, ^#^*P* < 0.1.

^*a*^n = 8-10 due to limited tissue amount.

## Discussion

This study presents repeated, integrated and comprehensive investigations during 2 years following RYGB in patients with type 2 diabetes and obesity. Our current results point to early effects, within weeks, involving autonomic nerve and cortisol axis activity and lowering of systemic inflammation. Later on, there are changes in adipose tissue gene expression and improved glucose handling. Accordingly, 2 years after RYGB, measures of insulin sensitivity, lipid levels, body composition, and adipocyte glucose uptake were similar to BMI-matched healthy control subjects. However, there was a slight remaining hyperglycemia and a lower adipocyte size. The effects likely contribute to the metabolic improvements during different phases after RYGB. The sustained and clinically important improvements in metabolic homeostasis found is in accordance with previous results of bariatric surgery (14,20,27). The very first, rapid glycemic improvement occurring within days is likely due to lowering of endogenous, mainly hepatic, glucose production (14).

The patients included in this study weighed less than the typical bariatric surgery patient, and they clearly benefit from RYGB surgery in terms of substantial reductions in body fat, glycated hemoglobin levels, and the need for glucose-lowering drugs. Indices of insulin resistance show significant improvements within a few months, and these remain relatively unchanged for at least 2 years. In contrast, beta-cell function measured as glucose-stimulated insulin secretion (insulinogenic index) did not change.

The reduced need for antihypertensive treatment indicates beneficial effects on blood pressure. The high frequency (HF; 0.12-0.40 Hz) component of HRV is considered to reflect parasympathetic activity, whereas the low frequency (0.04-0.12 Hz) component reflects the combined activity of the sympathetic and parasympathetic systems. Thus, the increased overall spectral power and increased HF power from 4 weeks together with a subsequently reduced low frequency to HF ratio at 24 weeks most likely indicates an increase of parasympathetic, also relative to sympathetic, tone, which partly resembles our findings during experimental hypoglycemia (17). These effects may be transient, since HRV measures returned toward baseline values at 104 weeks. There was however also a sustained and significant reduction of heart rate, and this may suggest a long-lasting change in the balance between sympathetic and parasympathetic activity.

A rapid and sustained reduction in WBC counts and CRP levels, albeit within the normal range at baseline, suggest a reduction of systemic inflammation, which could potentially contribute to some of the beneficial cardiovascular and metabolic effects of RYGB (3). However, we recently reported a remaining elevation of some inflammatory cytokines post-RYGB (28). We detected a significant early and transient decrease in morning serum cortisol at 4 weeks after surgery and a trend toward a sustained lowering of ACTH. While elevated cortisol levels are well known to be linked to the metabolic syndrome in disease states such as Mb Cushing, circulating cortisol has not been consistently found to correlate with weight or BMI in otherwise healthy obese subjects (29). Tissue-specific dysregulation of cortisol turnover had been suggested as an alternative route by which cortisol action may be affected in obesity, implicating differential regulation of the enzyme 11β-hydroxysteroid dehydrogenase type 1 (11-β-HSD1) that locally generates cortisol from cortisone (30). However, the observed changes in ACTH and cortisol levels in our study were not coupled with any changes in the adipose tissue expression of 11-β-HSD1. Few studies have specifically focused on the cortisol axis following bariatric surgery, and those that exist have rendered inconclusive results (14) as to whether there is a change with surgery and to the direction of change. Our results point toward modulating effects of RYGB on the hypothalamic-pituitary-adrenal axis, but larger studies are required to confirm this. While the exact mechanisms underlying the observed effects on ACTH/cortisol and ANS regulation are beyond the scope of this study, it is conceivable that changes in hemodynamics, neural input via vagal and sympathetic afferents, or brain sensing of glucose, other nutrients and hormones could contribute. On this matter, a very recently published study from our group highlights CNS effects following RYGB in nondiabetic individuals. It demonstrated changes in the regulation of the brain’s blood flow, glucose uptake, and activation of neural networks involving the striatum, thalamus, and hypothalamus (31).

IGF-1 levels have previously been reported as lower in obese than in normal-weight subjects (32), and an increase in IGF-1 levels, due to rising growth hormone levels, has previously been observed after bariatric surgery (21), consistent with the results of this study. Taken together, the data suggest that neuroendocrine pathways linking the central nervous system and peripheral metabolism are important for the favorable antidiabetic effects of RYGB, but this requires more research, for example, to establish causal *vs* adaptive relationships.

In this study, some adipose effects occurred rapidly, in particular a reduction of the individual adipocyte volume. This is most likely due to lipid depletion and redistribution toward other tissues such as liver and muscle to provide energy fuel (33). Such a lipid flux away from adipose tissue may in turn be caused by a catabolic condition where a reduction of calorie intake is a major contributing factor. In contrast, improved glucose uptake capacity of adipocytes was evident only at 2 years after RYGB. We previously reported that while LCD had no effect on cell morphology, adipocyte size was reduced at 4 weeks post-RYGB, despite comparable weight loss (20). Taken together, our data suggest that the rapid decrease in adipocyte size after RYGB surgery could be a feature that is specifically linked to the procedure, whereas later on the reduced adipocyte size is maintained due to sustained weight loss. Fat cell enlargement is an independent marker of insulin resistance (34); however, no significant change in adipocyte insulin sensitivity was seen at the 4-week visit despite the marked reduction in cell size. The increase in adipocyte glucose uptake seen at 104 weeks suggests that adipose tissue glucose utilization might increase at a later stage, as a secondary phenomenon. Furthermore, the glucose uptake at 104 weeks was comparable to that of BMI-matched nondiabetic individuals, thus reflecting long-term normalization of adipocyte function by RYGB. In agreement with our findings, Andersson et al (35) showed that a reduction in subcutaneous fat cell size 104 weeks after RYGB in obese women was more strongly associated with the improvement of insulin sensitivity than fat mass reduction per se. In line with these results, we observed that the expression of genes involved in glucose transport did not change significantly at 4 weeks but gradually increased at 24 and 104 weeks postsurgery. Of note, expression of genes involved in adipogenesis like *PPARG*, *CEBP*, *BMP4*, and *FAS* was gradually increased over time postsurgery, indicating the activation of adipogenic pathways may have contributed to a greater number of adipocytes and a smaller average size.

We did not find any significant change in adipocyte lipolysis after RYGB, whereas a previous study reported a reduced basal lipolysis (35), which may be supported by the lowering of fasting plasma glycerol and lower glycerol and FFA AUCs during OGTT at 104 weeks in our patients. However, we also found an increased adipose tissue expression of the *CPT1B* gene over time, suggesting that lipid oxidation may be enhanced. There are some previous data supporting an upregulation of lipid oxidation after gastric bypass procedures and that this may contribute to loss of body fat and weight reduction and maintenance (36). Our current data on the enzyme CPT-1 in adipose tissue, together with a stable lipolysis rate and adipocyte shrinkage (lipid depleted), are in agreement with this notion (37). Nonetheless, long-term depletion of lipid energy stores in adipose tissue will obviously require a negative energy balance that in turn requires a reduction of calorie intake, which is an important and necessary component of the profound weight loss achieved following RYGB (38).

Obesity-driven accumulation of excess lipids in adipocytes is accompanied by infiltration of immune cells and inflammation (39). Studies have reported that pro-inflammatory cytokines like interleukin 6 and interleukin 18 are elevated in obesity and that this occurs in parallel with changes to other inflammatory mediators as well as the development of insulin resistance (40-42). We found that adipose tissue expression of genes *IL6* and *IL18* was reduced post-RYGB, which is in agreement with some previous studies showing reduced plasma levels following weight loss (41,43). This was seen from 24 weeks, and thus anti-inflammatory effects in adipose tissue may occur later than, and potentially as a consequence of, reduced systemic inflammation since CRP and WBC had been markedly reduced already at 4 weeks. There was a reduction in the expression of resistin, an adipokine associated with adipocyte insulin resistance (44). Together with the lowering of WBC and CRP levels, these results point toward a reduction of adipose as well as systemic inflammatory activity after RYGB.

Surprisingly, the expression of *FKBP5*, *CNR1*, and *LCN2* genes were increased after surgery. The expression of these genes is known to be upregulated by glucocorticoids and is linked to insulin resistance in adipose tissue. Since cortisol levels were not elevated but rather decreased initially and there was no change in adipose gene expression of the enzyme 11-β-HSD1, it can be speculated that the sensitivity to cortisol was increased specifically in adipose tissue.

**Figure 5.**
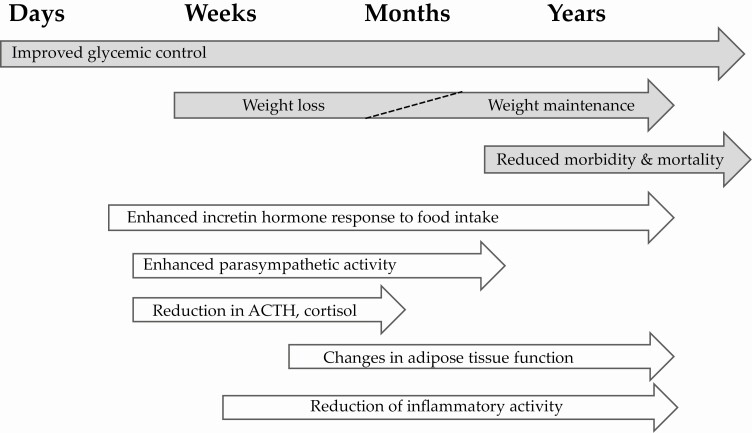
Schematic and hypothetical overview of changes after RYGB as they occur over time. Grey arrows, clinical effects; white arrows, effects on endocrine, autonomic nerve, and adipose functions, which may contribute to the favorable clinical effects.

As summarized in Figure 5, our results suggest the following course of events following RYGB in individuals with obesity and type 2 diabetes: (1) within days, a very rapid improvement in glycemic control, initially due to reduced hepatic glucose production; (2) within a few weeks, a partial loss of lipid content, a reduced average size of subcutaneous adipocytes, and an enhanced recruitment of new adipocytes with, in parallel, an increased insulin sensitivity and attenuation of cortisol axis activity together with increased parasympathetic tone; (3) within a few months, near-maximal effects on reduction of body weight, body fat, and inflammation and an increase in adipocyte glucose uptake and proliferation and whole-body insulin sensitivity; and (4) beyond 6 months, maintained body weight reduction and an insulin sensitivity and adipose glucose utilization similar to healthy BMI-matched individuals (albeit with a remaining mild hyperglycemia).

This study has some clear limitations. It is acknowledged that the results need further validation in a larger cohort since the number of participants was small, notwithstanding the detailed and repeated integrative assessments. Further, the presurgery routine LCD diet is expected to contribute to the effects demonstrated early postsurgery. We are currently investigating this in detail in a separate ongoing randomized trial directly comparing effects after 4 weeks of LCD to those at 4 weeks after RYGB without diet pretreatment.

We conclude that, besides the well-established changes in gut hormones, neuroendocrine factors involving the ANS and cortisol axis are likely to contribute to the rapid glycemic improvement after RYGB. There are also signs of an early reduction of low-grade inflammation. Thereafter, effects on metabolic functions and gene expression in adipose tissue may further improve and maintain long-term insulin sensitivity and glucose and lipid homeostasis. Such effects are largely independent of the magnitude of weight loss as they occur early (days to weeks); in addition, the sustained metabolic effects (years) are not accounted for solely by weight loss per se. We propose that following RYGB sequential changes in neuroendocrine pathways including the cortisol axis and the ANS, systemic inflammation, and, later on, adipose tissue function are important for the beneficial short- and long-term outcomes, such as remission of diabetes and prevention of its microvascular and cardiovascular comorbidities. Importantly, neuroendocrine alterations that are partly opposite to those reported here may contribute to the development of type 2 diabetes (45) and therefore a detailed understanding of RYGB effects may provide novel concepts for pharmacological or lifestyle interventions to halt diabetes development and progression.

## Data Availability

Data and study protocol can be made available by the authors upon request.

## Additional Information

***Disclosures:*** The authors have no potential conflict of interest to declare.
